# Supplementary material for: Deep molecular profiling of synovial biopsies in the STRAP trial identifies signatures predictive of treatment response to biologic therapies in rheumatoid arthritis
Source: Nat Commun. 2025 Jul 2;16:5374. doi: 10.1038/s41467-025-60987-9 (PMC12223067; doi:10.1038/s41467-025-60987-9)
Supplement: Supplementary file 1 — Supplementary Information [file 41467_2025_60987_MOESM1_ESM.pdf]

Supplementary Material

Deep molecular profiling of synovial biopsies in the STRAP trial identifies signatures predictive of treatment response to biologic therapies in Rheumatoid Arthritis

Myles J. Lewis,* Cankut Çubuk, Anna Surace, Elisabetta Sciacca, Rachel Lau, Katriona Goldmann, Giovanni Giorli, Liliane Fossati-Jimack, Alessandra Nerviani, Felice Rivellese, Costantino Pitzalis* and the STRAP collaborative group

*Co-senior authors

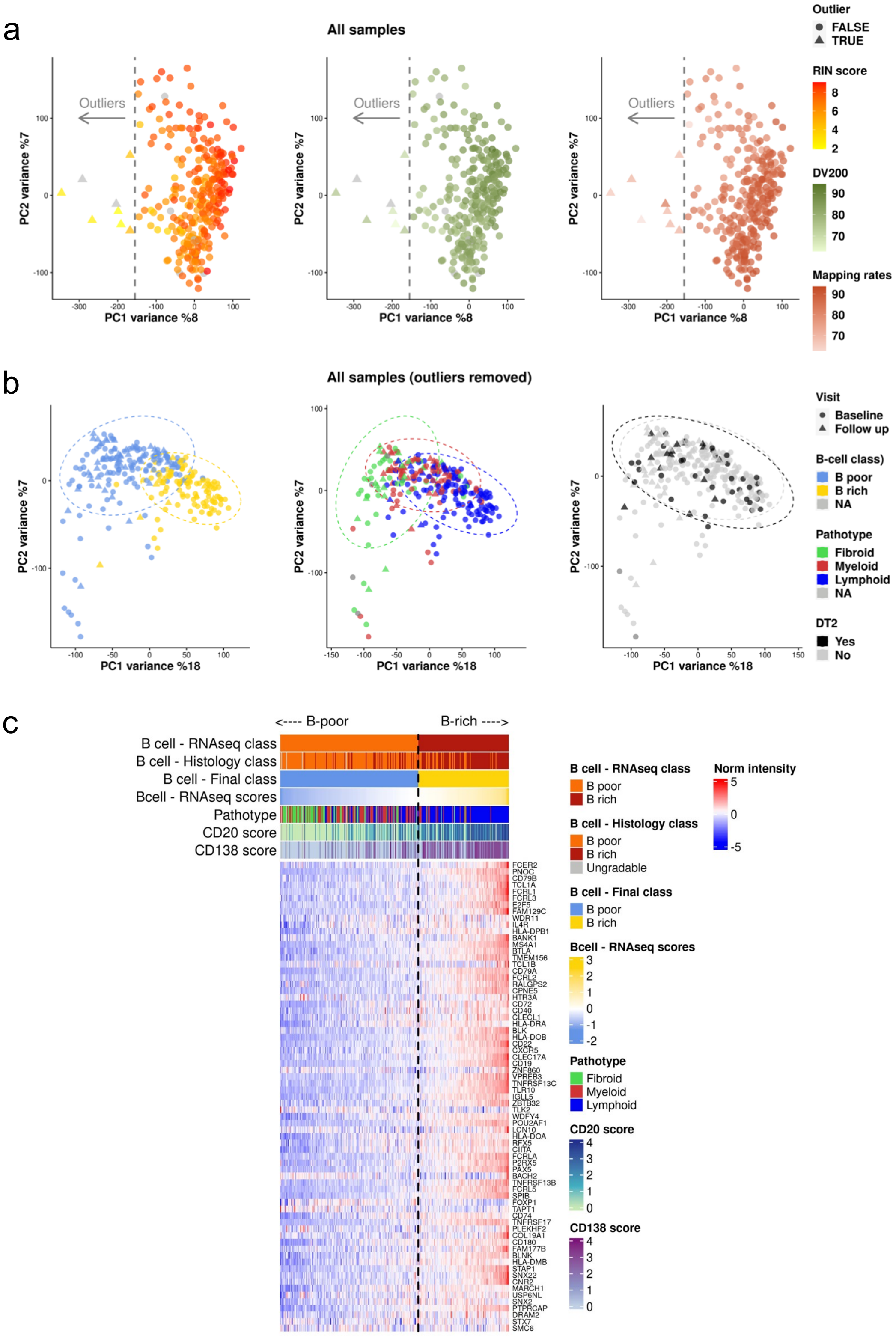

Supplementary Figure 1. Unsupervised projection of RNA-Seq for data quality control
a, Two-dimensional representation of RNA-Seq data displays outlier samples and explains their association with low RNA quality and consequently with low mapping rates. **b,** PCA of synovial transcriptome showed a clear separation between B-cell rich and poor groups. Similar clustering was also observed for pathotypes where lympho-myeloid and fibroid/pauci-immune groups formed clear clusters, whereas the diffuse-myeloid partially overlapped with these two groups. **c,** Expression profile of the gene set used to calculate B-cell module scores and to generate B-cell rich/poor categories. Column annotation tracks show B-cell module score and cell type (B cell rich/poor) quantified using both RNA-Seq and histopathology data, pathotype classification and semi-quantitative histological scores of pathotype markers (CD20 and CD138).

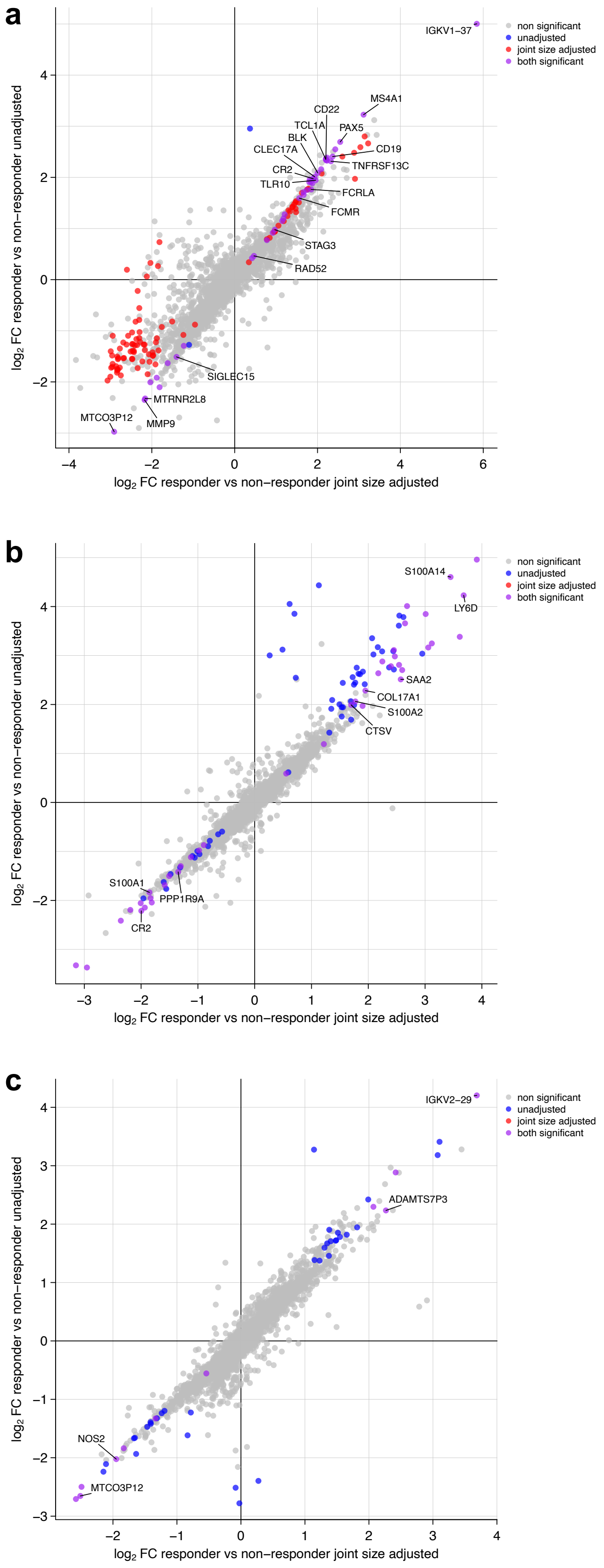

Supplementary Figure 2. Differential gene expression analysis with or without adjustment for joint size as covariate
Scatter plots showing $\log_2$ fold change for differential expression analysis between responders and non-responders adjusted for joint size on *x* axis and unadjusted fold change on *y* axis for individuals treated with **a,** etanercept, **b,** tocilizumab or **c,** rituximab. Statistical analysis by negative binomial distribution generalised linear regression on count data via DESeq2. P-values were calculated by two-sided Wald test with FDR correction (Benjamini-Hochberg) for multiple testing.

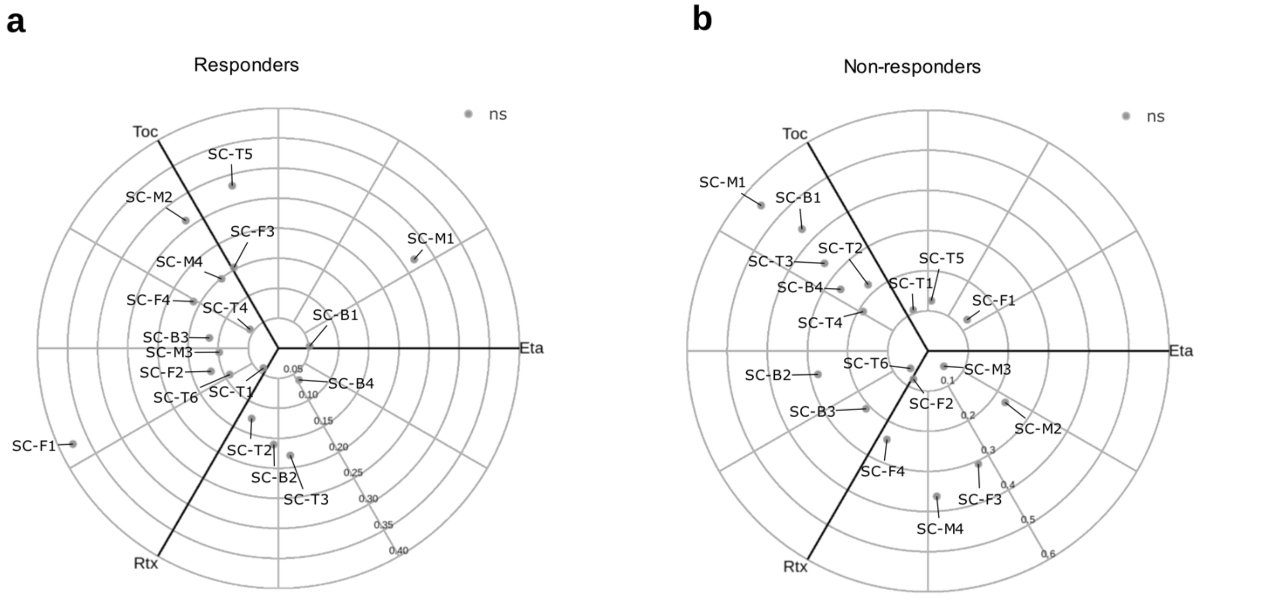

Supplementary Figure 3. The three-way comparison for cell subtypes
a, Three-way polar plot comparing AMP single-cell subclass levels in responders across three treatment groups **b,** result for the non-responders. Statistical analysis by one-way ANOVA, with two-sided pairwise t-tests. $P < 0.05$ was considered statistically significant for both comparisons.

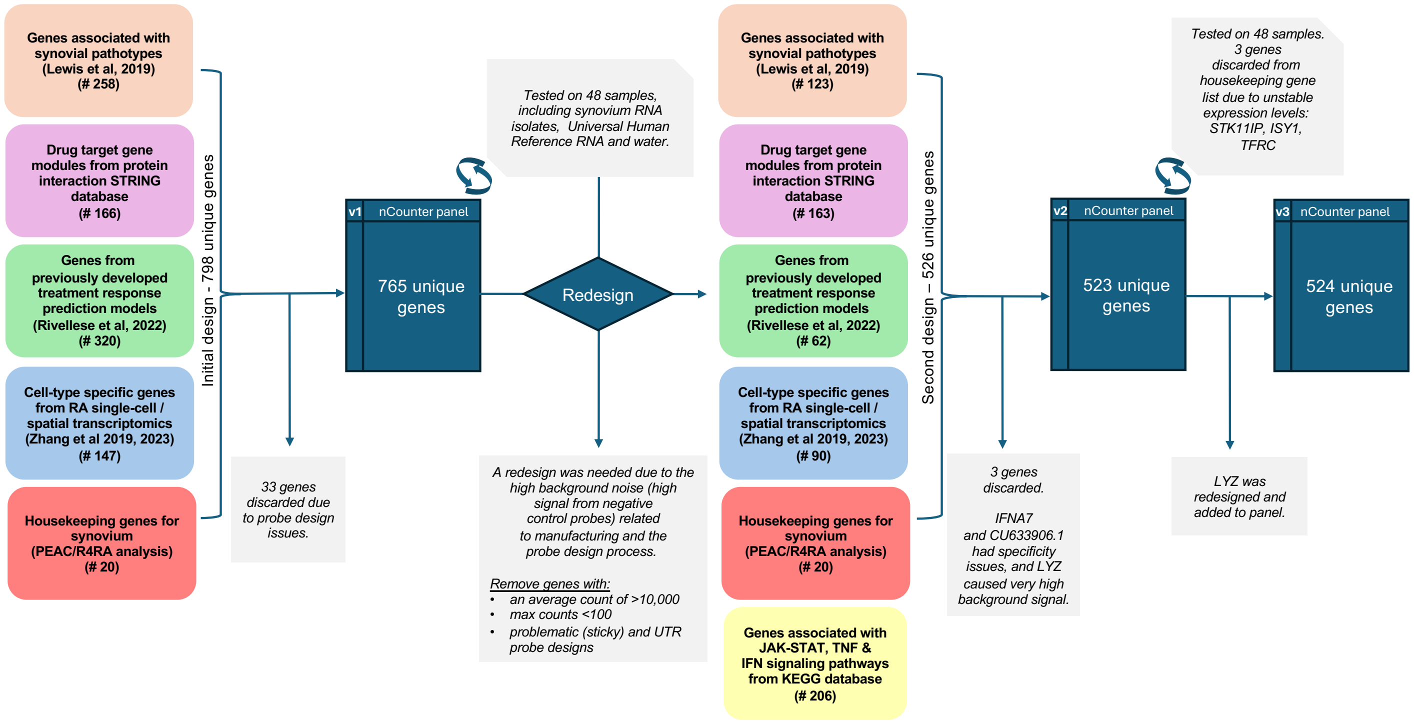

Supplementary Figure 4. Illustration of the steps involved in the creation of the custom synovium nCounter panel
A custom panel was developed in two main stages. 765 genes were included in the first version, derived from five subpanels. The test assay results on 48 synovial biopsy samples showed that negative control probes were generating stronger signals than anticipated compared to some gene probes (i.e. high background noise). Through collaboration with scientists from Nanostring, in the second version the total number of genes used in the panel was pared down, with less informative genes with either excessively high expression or very low expression being removed. Redesign of selected problematic gene probes was performed when possible. Thus for the second stage, 524 genes from six subpanels were included. Test runs of version 2 onwards did not report abnormal signals from control probes, and the final version was manufactured for this study.

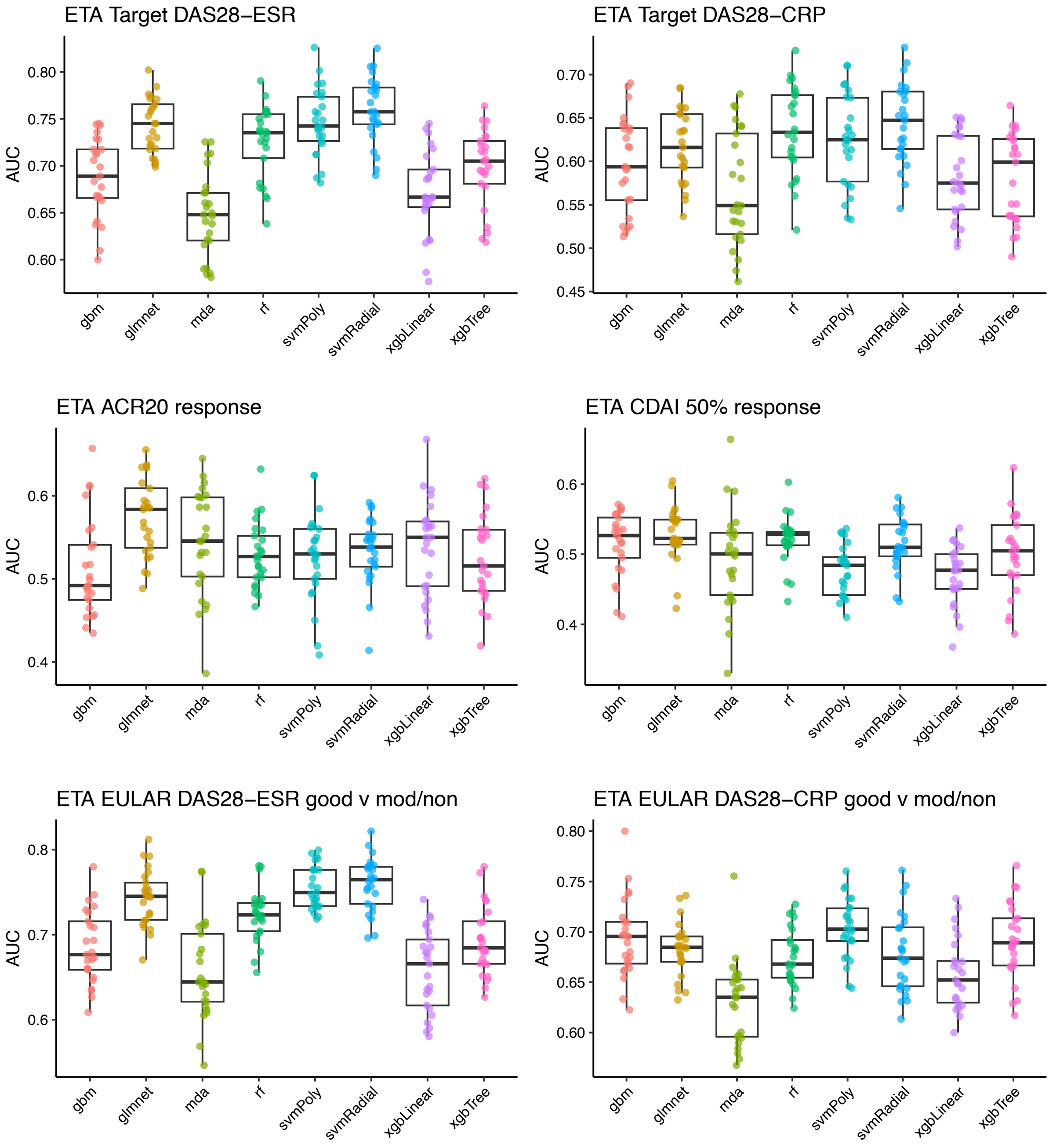

Supplementary Figure 5. Machine learning models comparing different response outcomes following Etanercept
25× repeated 10×10-fold nested cross-validation applied to machine learning models for a) etanercept, b) tocilizumab c) rituximab to identify which response outcome resulted in the best predictive models. Model performance for each repeat was measured by area under receiver operating characteristic (ROC) curve (AUC) analysis. Model types: gradient boosted machine (gbm), elastic net regression (glmnet), mixed discriminant analysis (mda), random forest (rf), support vector machine (svm) with polynomial (svmPoly) or radial (svmRadial) kernel, extreme gradient boosting (xgboost) with tree booster (xgbTree) or linear booster (xgbLinear). Box plots show median, upper and lower quartiles, with whiskers denoting maximal and minimal data within 1.5 × interquartile range.

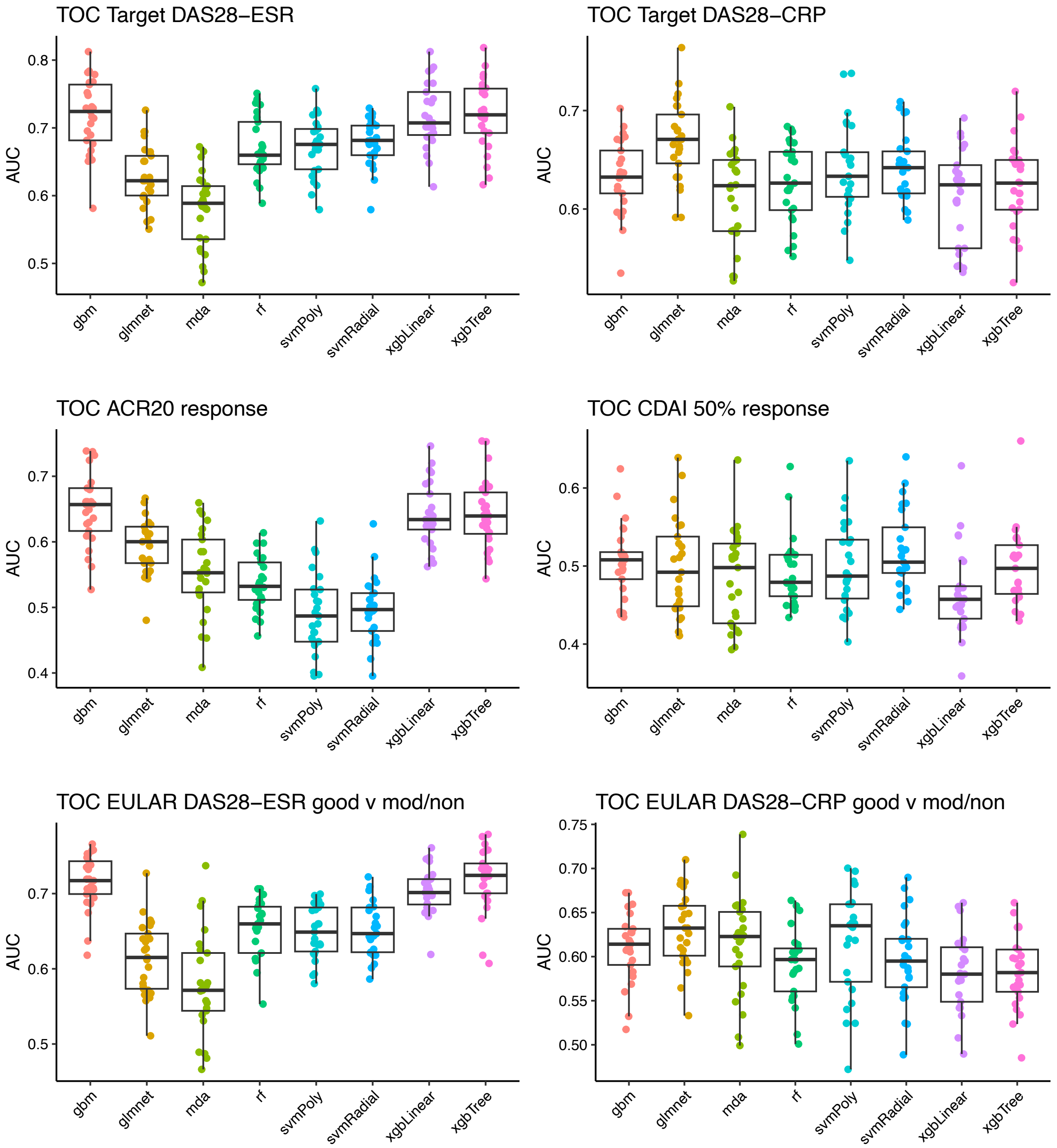

Supplementary Figure 6. Machine learning models comparing different response outcomes following Tocilizumab
25× repeated 10×10-fold nested cross-validation applied to machine learning models for a) etanercept, b) tocilizumab c) rituximab to identify which response outcome resulted in the best predictive models. Model performance for each repeat was measured by area under receiver operating characteristic (ROC) curve (AUC) analysis. Model types: gradient boosted machine (gbm), elastic net regression (glmnet), mixed discriminant analysis (mda), random forest (rf), support vector machine (svm) with polynomial (svmPoly) or radial (svmRadial) kernel, extreme gradient boosting (xgboost) with tree booster (xgbTree) or linear booster (xgbLinear). Box plots show median, upper and lower quartiles, with whiskers denoting maximal and minimal data within 1.5 × interquartile range.

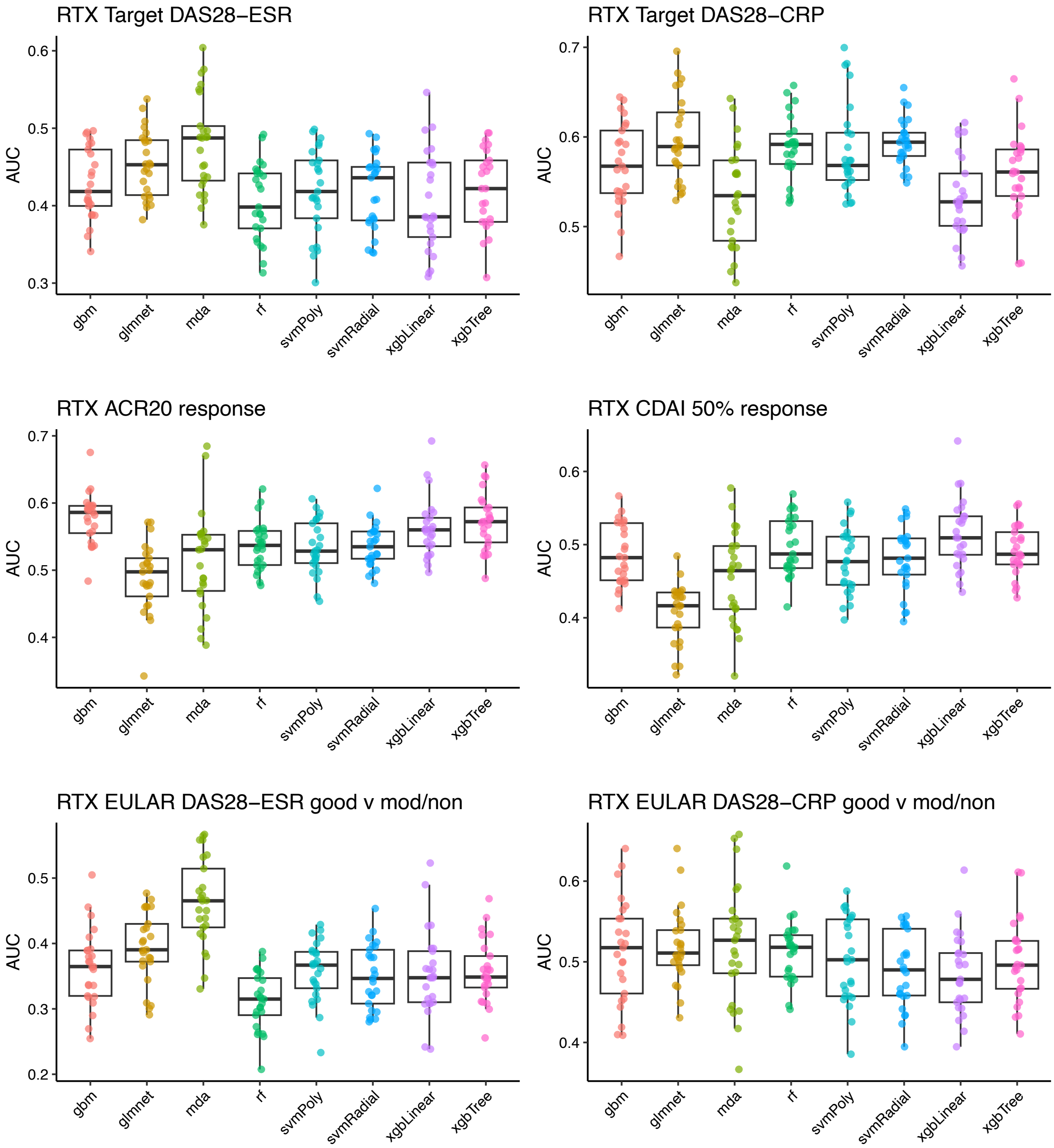

Supplementary Figure 7. Machine learning models comparing different response outcomes following Rituximab
25× repeated 10×10-fold nested cross-validation applied to machine learning models for a) etanercept, b) tocilizumab c) rituximab to identify which response outcome resulted in the best predictive models. Model performance for each repeat was measured by area under receiver operating characteristic (ROC) curve (AUC) analysis. Model types: gradient boosted machine (gbm), elastic net regression (glmnet), mixed discriminant analysis (mda), random forest (rf), support vector machine (svm) with polynomial (svmPoly) or radial (svmRadial) kernel, extreme gradient boosting (xgboost) with tree booster (xgbTree) or linear booster (xgbLinear). Box plots show median, upper and lower quartiles, with whiskers denoting maximal and minimal data within 1.5 × interquartile range.

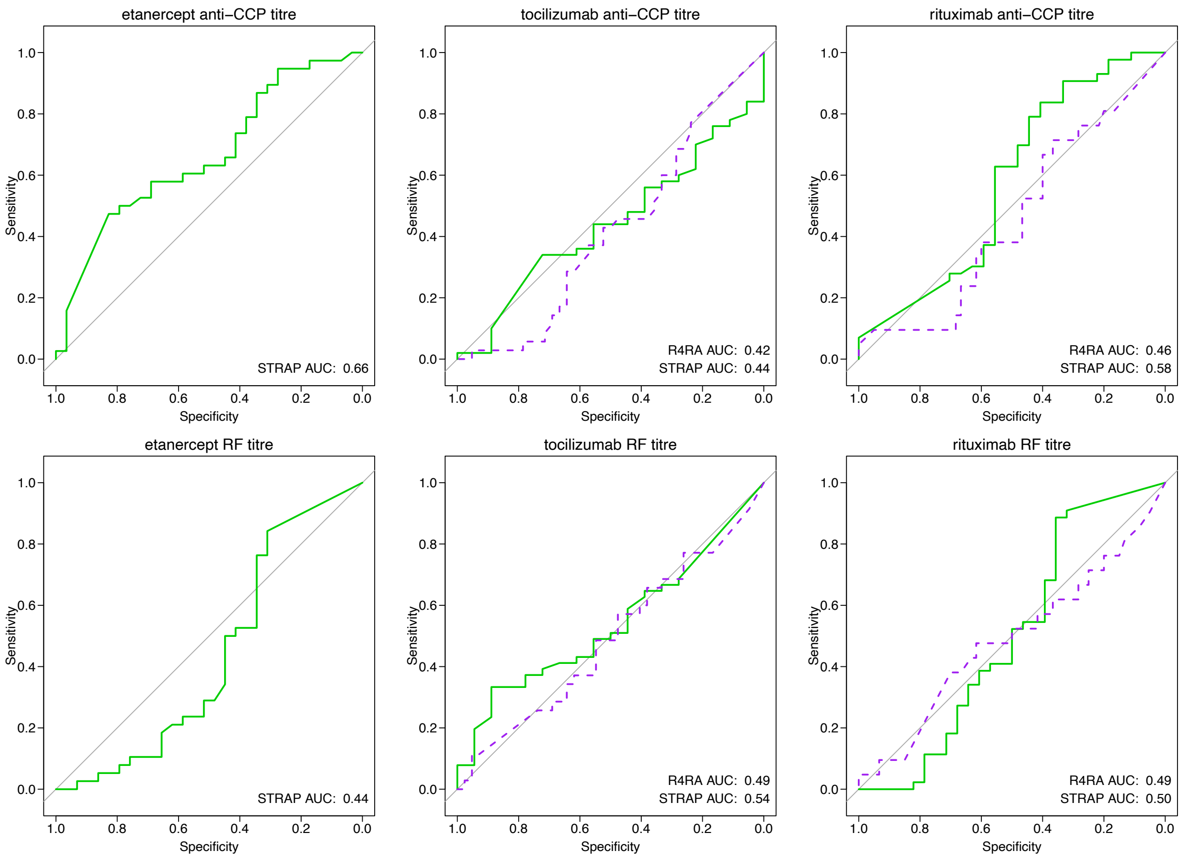

Supplementary Figure 8. ROC curves showing the performance of anti-CCP titre and rheumatoid factor titre for prediction of response after 16 weeks of biologic therapy in STRAP and R4RA

Patients in the STRAP trial were randomised to etanercept, tocilizumab or rituximab. Patients in the R4RA trial were randomised to tocilizumab or rituximab only.

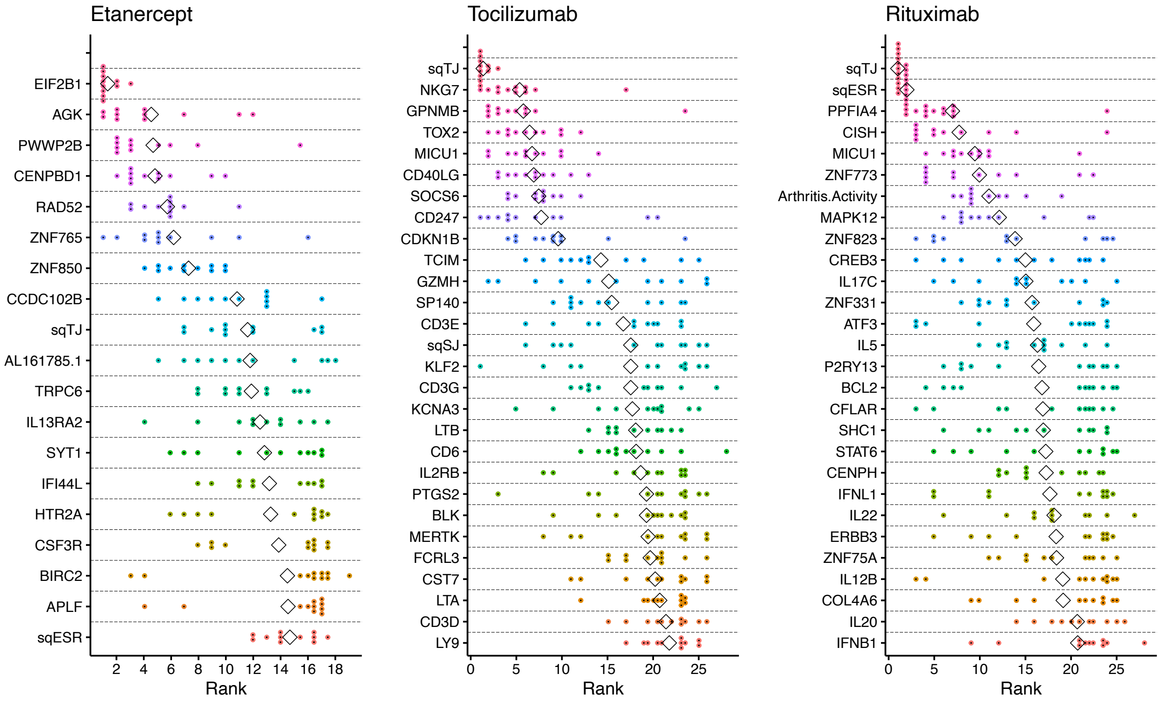

Supplementary Figure 9. Variable rank plots for machine learning models predicting response
Rankings for predictors from machine learnings for predicting target DAS28-ESR response at 16 weeks for each randomised drug. Predictors from the best model across each outer CV fold as well as the final model fitted on the whole data were ranked in order of variable importance. Points show the distribution of ranks across outer CV folds and the final model. Diamonds show mean rank.

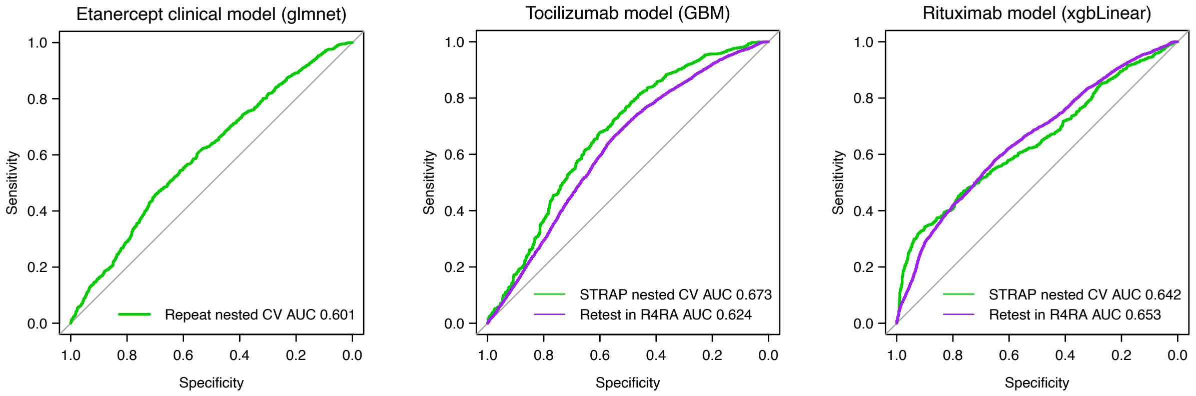

Supplementary Figure 10. ROC curves for machine learning models built using baseline clinical parameters alone
Machine learning models were built using the same input baseline clinical parameters as for the full models including gene expression. Repeated nested 10x10-fold CV was performed to estimate model performance with 25 repeats. ROC curves show AUC over the 25 repeats for predicting target DAS28-ESR response at 16 weeks for each drug. The etanercept model was tested in STRAP alone as no etanercept data is available in the R4RA trial. The repeated nested outer-fold CV models generated in STRAP and tested in STRAP by nested 10x10-fold CV, were then additionally tested in R4RA for patients treated with tocilizumab and rituximab.

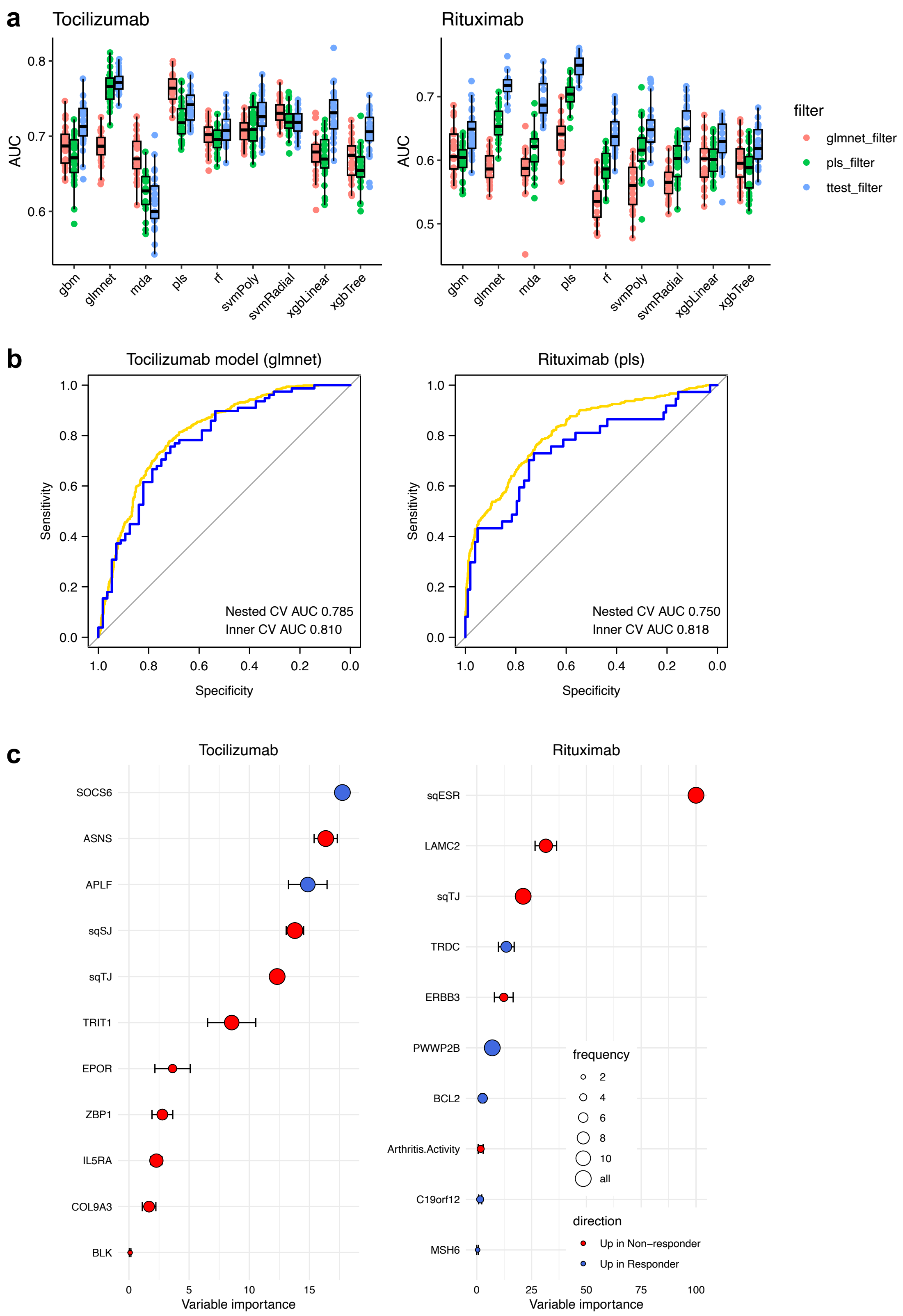

Supplementary Figure 11. Tocilizumab and rituximab predictive models trained on merged STRAP and R4RA RNA-Seq data and clinical parameters

RNA-Seq data from STRAP and R4RA were merged. Predictive models were built using 10x10-fold nested CV with 25 repeats. a) Performance of machine learning models measured by ROC AUC using repeated nested CV. Box plots show median, upper and lower quartiles, with whiskers denoting maximal and minimal data within 1.5 × interquartile range. b) ROC curves for the best and final models for each drug (glmnet model with t-test filter for tocilizumab; PLS model with t-test filter for rituximab) trained on merged STRAP and R4RA data showing ROC curves for nested CV test folds as well as inner CV test folds. c) Variable importance and stability plots showing variation in variable importance across outer CV folds. Error bars show standard error of mean variable importance.

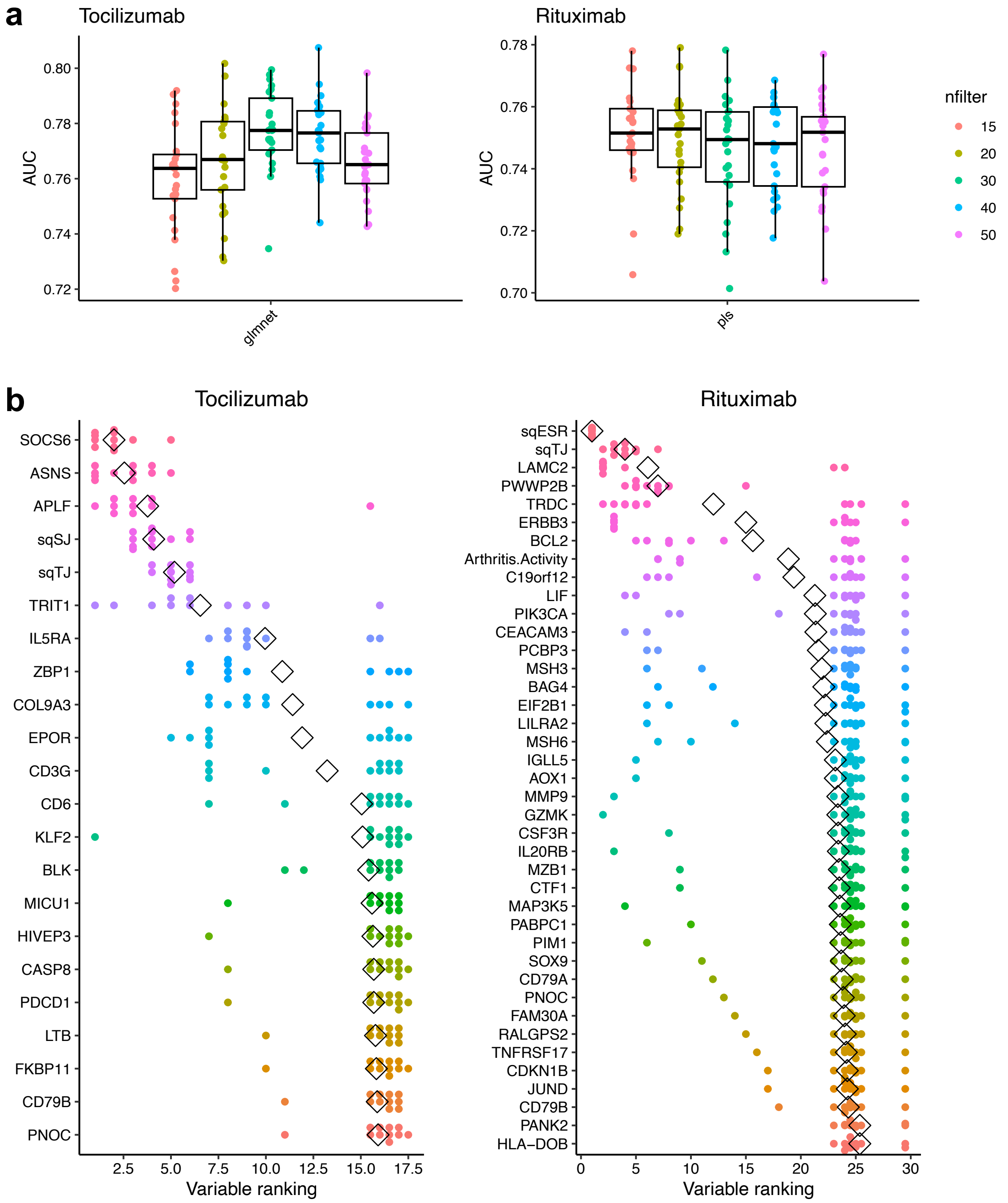

Supplementary Figure 12. Model tuning plots and variable ranking plots for models trained on merged STRAP and R4RA RNA

a) Model tuning plots from repeated nested CV showing optimisation of number of parameters selected by feature filters. Box plots show median, upper and lower quartiles, with whiskers denoting maximal and minimal data within 1.5 × interquartile range. b) Variable ranking plots showing ranking of variables across outer CV folds. Diamonds show mean rank.

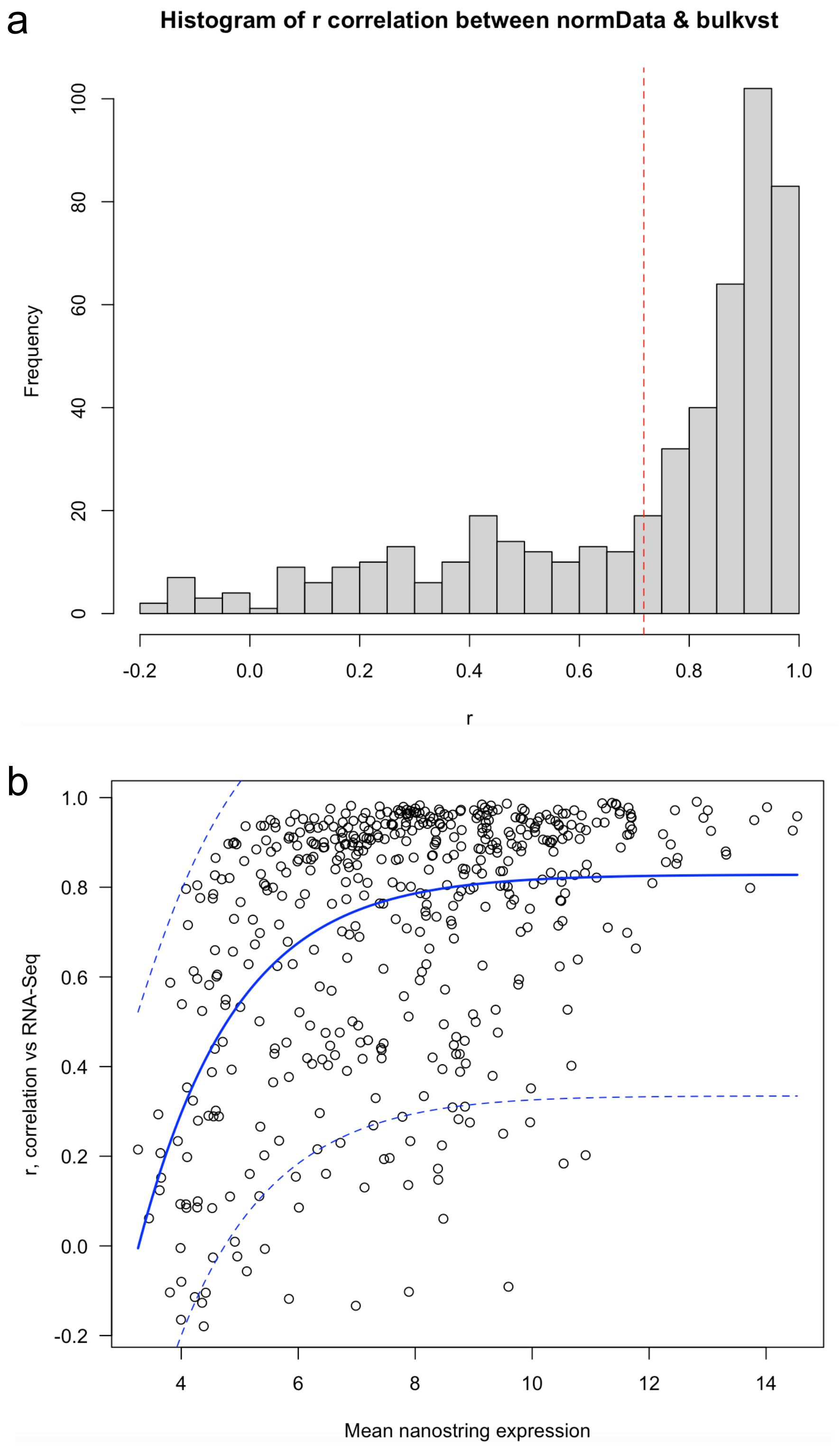

Supplementary Figure 13. Correlation between nCounter and RNA-Seq applied to synovial biopsies from STRAP
a, Frequency histogram for Pearson correlation coefficients for each gene comparing correlation between nCounter and bulk RNA-Seq applied to synovial biopsies from the STRAP cohort. Median Pearson correlation coefficient is shown by dashed red line, showing that the majority of genes show high levels of correlation with RNA-Seq. **b,** Scatter plot showing that correlation between nCounter and RNA-Seq tends to decrease when mean nCounter expression is low.

Supplementary Table 1: Baseline characteristics of patients with available RNA-seq

	Overall (n=208)	Etanercept (n=67)	Rituximab (n=72)	Tocilizumab (n=69)	Unadjusted p value
**Gender (Male)**	49 (24%)	16 (24%)	16 (22%)	17 (25%)	0·94
**Age, years**	53·4 (13·6)	51·9 (13·3)	55·0 (13·5)	53·3 (14·0)	0·41
**Disease Duration, years**	4·0 [1·0, 8·0]	5·0 [2·0, 9·0]	3·0 [1·0, 8·0]	3·0 [1·0, 8·0]	0·31
**Study (UK)**	173 (83%)	57 (85%)	58 (81%)	58 (84%)	0·75
**Clinical disease activity index (CDAI)**	33·8 [25·9, 43·1]	34·7 [26·5, 44·4]	31·4 [26·0, 40·8]	34·0 [25·8, 42·0]	0·53
**Erythrocyte sedimentation rate (ESR), mm/h**	27·0 [16·0, 42·0]	24·0 [14·0, 37·0]	27·0 [16·0, 45·0]	28·0 [15·0, 45·0]	0·25
**C-reactive protein (CRP), mg/L**	11·5 [3·6, 27·0]	9·5 [3·2, 24·8]	11·0 [4·0, 29·5]	13·0 [4·0, 28·0]	0·32
**Rheumatoid factor (RF) OR Anti–citrullinated protein antibody (ACPA) positive**	179 (86%)	59 (88%)	64 (89%)	56 (81%)	0·35
**Rheumatoid factor (RF) positive**	146 (70%)	48 (72%)	52 (72%)	46 (67%)	0·73
**Anti–citrullinated protein antibody (ACPA) positive**	167 (80%)	53 (79%)	60 (83%)	54 (78%)	0·72
**Creatinine (μmol/L)**	62·0 [54·0, 70·0]	61·0 [56·0, 70·0]	62·0 [52·0, 69·0]	60·0 [54·0, 68·0]	0·95
**Alanine aminotransferase (ALT), U/L**	17·0 [13·0, 22·0]	17·0 [13·5, 22·0]	18·0 [14·0, 27·0]	16·5 [13·0, 21·8]	0·75
**Aspartate aminotransferase (AST), U/L**	21·0 [17·0, 24·8]	21·5 [18·0, 24·0]	22·0 [16·5, 28·0]	19·0 [17·0, 23·0]	0·45
**Haemoglobin, g/L**	120·5 [111·8, 130·8]	125·5 [119·0, 130·0]	123·5 [117·8, 135·5]	113·0 [101·2, 120·5]	0·006
**White Blood Cell count, 10^9/L**	7·6 (2·3)	7·5 (2·5)	8·5 (2·2)	7·1 (1·9)	0·1
**Platelets, 10^9/L**	293·0 [245·5, 337·5]	277·0 [245·8, 304·8]	296·0 [234·5, 323·8]	313·0 [268·8, 376·8]	0·16
**Neutrophils, 10^9/L**	4·8 [3·6, 6·3]	4·6 [3·3, 6·5]	5·0 [4·2, 6·5]	4·7 [3·4, 5·5]	0·44
**Lymphocytes, 10^9/L**	1·6 [1·3, 2·0]	1·4 [1·2, 1·8]	1·9 [1·5, 2·2]	1·6 [1·3, 2·0]	0·13
**Synovial semi-quantitative scores**					
**CD20**	1·0 [0·0, 3·0]	1·0 [0·0, 3·0]	2·0 [0·0, 3·0]	1·0 [0·0, 3·0]	0·49
**CD138**	1·0 [0·0, 3·0]	1·0 [0·0, 2·2]	1·0 [0·0, 2·0]	2·0 [0·0, 3·0]	0·35
**CD68 lining**	1·0 [0·0, 2·0]	1·0 [0·0, 1·0]	1·0 [0·0, 2·0]	1·0 [0·0, 2·0]	0·86
**CD68 sub-lining**	1·0 [0·0, 2·0]	1·0 [0·0, 2·0]	1·0 [0·8, 2·0]	1·0 [1·0, 2·0]	0·55
**CD3**	1·0 [1·0, 2·0]	1·0 [0·8, 2·0]	1·5 [1·0, 2·0]	1·0 [1·0, 2·0]	0·25
**CD21 (Positive)**	8 (7%)	4 (12%)	2 (5%)	2 (5%)	0·45

**Synovial Pathotype**					0·95
**Fibroid**	58 (28%)	19 (30%)	21 (29%)	18 (26%)	
**Myeloid**	34 (17%)	12 (19%)	11 (15%)	11 (16%)	
**Lymphoid**	113 (55%)	33 (52%)	40 (56%)	40 (58%)	
**B cell status**					0·25
**B cell poor**	113 (54%)	39 (58%)	40 (56%)	34 (49%)	
**B cell rich**	95 (46%)	28 (42%)	32 (44%)	35 (51%)	
**Joint size**					0·24
**Small**	150 (72%)	49 (73%)	56 (78%)	45 (65%)	
**Large**	58 (28%)	18 (27%)	16 (22%)	24 (35%)	
**Number of tender joints, 0-28**	12·0 [7·0, 19·0]	14·0 [8·5, 20·0]	10·0 [7·0, 18·0]	12·0 [7·0, 19·0]	0·37
**Number of swollen joints, 0-28**	7·0 [4·0, 11·0]	7·0 [4·0, 10·5]	7·0 [4·0, 11·0]	7·0 [5·0, 10·0]	0·93
**28 joint count Disease Activity Score (DAS-28), ESR**	6·0 [5·3, 6·6]	6·0 [5·4, 6·5]	6·0 [5·2, 6·5]	6·0 [5·2, 6·9]	0·94
**28 joint count Disease Activity Score (DAS-28), CRP**	5·5 (1·1)	5·6 (1·0)	5·4 (1·1)	5·6 (1·2)	0·74
**Patient's global assessment, 0–100 VAS**	74·0 [55·0, 88·0]	73·0 [61·5, 88·0]	72·0 [46·0, 85·0]	77·0 [54·0, 89·0]	0·57
**Physician's global assessment, 0–100 VAS**	62·0 [44·0, 78·0]	67·0 [48·0, 79·5]	56·0 [38·0, 74·0]	60·0 [46·0, 83·0]	0·079
**Patient's assessment of tiredness, 0–100 VAS**	71·0 [52·0, 85·0]	75·0 [59·0, 85·5]	70·0 [49·0, 84·0]	68·0 [47·0, 85·0]	0·16
**Patient's assessment of pain, 0–100 VAS**	73·0 [56·0, 85·0]	73·0 [61·0, 84·5]	68·0 [46·0, 84·0]	76·0 [59·0, 88·0]	0·25
**HAQ total score**	1·6 [1·1, 2·1]	1·8 [1·1, 2·1]	1·6 [1·1, 2·1]	1·8 [1·1, 2·2]	0·56
**Functional Assessment of Chronic Illness Therapy (FACIT) score**	22·0 [13·0, 32·0]	21·0 [11·0, 32·0]	22·0 [15·0, 31·0]	24·0 [14·0, 32·0]	0·67
**Previous Methotrexate use**	129 (62%)	36 (54%)	49 (68%)	44 (64%)	0·21
**Previous Prednisolone use**	34 (16%)	12 (18%)	15 (21%)	7 (10%)	0·21
**Number of additional DMARDs used**					0·87
**1**	67 (32%)	20 (30%)	28 (39%)	19 (28%)	NA
**2**	99 (48%)	33 (49%)	30 (42%)	36 (52%)	NA
**3+**	39 (19%)	13 (19%)	13 (18%)	13 (19%)	NA

Data are n (%), median [IQR], mean (SD). CDAI=Clinical disease activity index. DAS28 =28 joint count disease activity score. CRP=C-reactive protein. ESR=erythrocyte sedimentation rate. P values are obtained using a two-sided Chi-Square test for categorical and binary variables, whereas a two-sided Student's T test or Mann-Whitney U test (based on normality, tested through Shapiro-Wilk test) was used for continuous variables. No adjustment for multiplicity was applied.

Supplementary Table 2. Contingency tables comparing joint size against clinical response at 16 weeks

i) ACR20 response

**Drug**	**Etanercept**		**Tocilizumab**		**Rituximab**	
**Response**	Non Response	Response	Non Response	Response	Non Response	Response
**Small joint**	20	29	12	33	21	35
**Large joint**	9	9	6	18	7	9
**Fisher test**	p-value = 0.58		p-value = 1		p-value = 0.77	

ii) DAS28-ESR < 3.2

**Drug**	**Etanercept**		**Tocilizumab**		**Rituximab**	
**Response**	Non Response	Response	Non Response	Response	Non Response	Response
**Small joint**	24	25	15	30	40	16
**Large joint**	9	9	6	18	11	5
**Fisher test**	p-value = 1		p-value = 0.5869		p-value = 1	

Statistical analysis by two-sided Fisher exact test. No adjustments were made for multiple testing.

Supplementary Table 3. Demographics table of study cohort stratified by unsupervised molecular clustering

	cluster 1 (N=37)	cluster 2 (N=75)	cluster 3 (N=96)	Total (N=208)	p value
**Age (years)**	53.4 (14.9)	53.9 (14.1)	53.0 (12.7)	53.4 (13.6)	0.92
**Gender**					0.4
F	31 (83.8%)	54 (72.0%)	74 (77.1%)	159 (76.4%)	
M	6 (16.2%)	21 (28.0%)	22 (22.9%)	49 (23.6%)	
**CCP titre**	116.1 (123.5)	187.1 (176.9)	215.6 (168.2)	187.6 (167.6)	0.0085
**CRP (ug/mL)**	29.5 (28.3)	15.8 (23.7)	22.7 (35.4)	21.4 (30.7)	0.073
**ESR (mm/hr)**	32.3 (20.5)	25.3 (19.2)	34.7 (21.8)	30.9 (21.0)	0.012
**HAQ Score**	1.8 (0.6)	1.5 (0.7)	1.6 (0.7)	1.6 (0.7)	0.18
**Cell type**					2.4e-09
B cell poor	18 (48.6%)	57 (79.2%)	30 (31.2%)	105 (51.2%)	
B cell rich	19 (51.4%)	15 (20.8%)	66 (68.8%)	100 (48.8%)	
**Serology**					0.0044
High positive RF or ACPA	22 (59.5%)	49 (65.3%)	82 (85.4%)	153 (73.6%)	
low positive RF or ACPA	6 (16.2%)	8 (10.7%)	6 (6.2%)	20 (9.6%)	
Negative RF / ACPA	9 (24.3%)	18 (24.0%)	8 (8.3%)	35 (16.8%)	
**RF**					0.036
negative	11 (29.7%)	30 (40.0%)	21 (21.9%)	62 (29.8%)	
positive	26 (70.3%)	45 (60.0%)	75 (78.1%)	146 (70.2%)	
**CD138 level**					4.2e-05
high	17 (45.9%)	18 (25.0%)	57 (59.4%)	92 (44.9%)	
low	20 (54.1%)	54 (75.0%)	39 (40.6%)	113 (55.1%)	
**Pathotype**					5e-11
Ungraded	0 (0.0%)	3 (4.0%)	0 (0.0%)	3 (1.4%)	
Fibroid	2 (5.4%)	28 (37.3%)	4 (4.2%)	34 (16.3%)	
Myeloid	13 (35.1%)	24 (32.0%)	21 (21.9%)	58 (27.9%)	
Lymphoid	22 (59.5%)	20 (26.7%)	71 (74.0%)	113 (54.3%)	
**DAS28 EULAR**					0.0059
Good Responder	20 (54.1%)	35 (46.7%)	49 (51.0%)	104 (50.0%)	
Moderate Responder	16 (43.2%)	22 (29.3%)	40 (41.7%)	78 (37.5%)	
Non Responder	1 (2.7%)	18 (24.0%)	7 (7.3%)	26 (12.5%)	

Data are represented as sample size (%) for categorical and binary variables and mean (SD) for numeric variables. CCP=anti-cyclic citrullinated peptide antibody, CRP=C-reactive protein, ESR=erythrocyte sedimentation rate, HAQ=health assessment questionnaire, RF=rheumatoid factor, ACPA=anti-citrullinated protein autoantibodies, DAS28=28-joint count disease activity score. P values were obtained using a two-sided Chi-Square test for categorical variables. Two-sided ANOVA F-test was used to compare continuous variables.

Supplementary Table 4. Results of machine learning from 10x10-fold nested CV with 25 repeats

Etanercept

Model	AUC	AUC.PR	Accuracy	Balanced accuracy	F1 Non-responder	F1 Responder	MCC
gbm	0.679 ± 0.0092	0.628 ± 0.0093	0.641 ± 0.0094	0.641 ± 0.0094	0.642 ± 0.0098	0.638 ± 0.0102	0.283 ± 0.019
glmnet	0.756 ± 0.0076	0.712 ± 0.0085	0.703 ± 0.0071	0.703 ± 0.0071	0.697 ± 0.0080	0.709 ± 0.0066	0.407 ± 0.014
mda	0.657 ± 0.0090	0.620 ± 0.0106	0.634 ± 0.0085	0.634 ± 0.0085	0.630 ± 0.0086	0.636 ± 0.0098	0.269 ± 0.017
rf	0.721 ± 0.0085	0.676 ± 0.0095	0.674 ± 0.0061	0.674 ± 0.0061	0.672 ± 0.0061	0.675 ± 0.0070	0.349 ± 0.012
svmPoly	0.750 ± 0.0076	0.716 ± 0.0086	0.690 ± 0.0093	0.690 ± 0.0093	0.681 ± 0.0096	0.698 ± 0.0098	0.381 ± 0.019
svmRadial	0.755 ± 0.0072	0.718 ± 0.0085	0.680 ± 0.0092	0.680 ± 0.0091	0.673 ± 0.0092	0.686 ± 0.0094	0.360 ± 0.018
xgbLinear	0.656 ± 0.0100	0.612 ± 0.0113	0.624 ± 0.0094	0.624 ± 0.0095	0.625 ± 0.0102	0.622 ± 0.0095	0.249 ± 0.019
xgbTree	0.680 ± 0.0082	0.634 ± 0.0102	0.648 ± 0.0084	0.649 ± 0.0085	0.652 ± 0.0089	0.643 ± 0.0091	0.299 ± 0.017

Tocilizumab

Model	AUC	AUC.PR	Accuracy	Balanced accuracy	F1 Non-responder	F1 Responder	MCC
gbm	0.718 ± 0.0107	0.834 ± 0.0062	0.714 ± 0.0067	0.622 ± 0.0085	0.449 ± 0.0149	0.807 ± 0.0047	0.273 ± 0.018
glmnet	0.629 ± 0.0089	0.759 ± 0.0076	0.681 ± 0.0074	0.596 ± 0.0066	0.420 ± 0.0097	0.780 ± 0.0060	0.210 ± 0.016
mda	0.579 ± 0.0112	0.691 ± 0.0073	0.623 ± 0.0091	0.568 ± 0.0096	0.407 ± 0.0131	0.722 ± 0.0076	0.133 ± 0.019
rf	0.672 ± 0.0087	0.792 ± 0.0056	0.685 ± 0.0064	0.613 ± 0.0077	0.451 ± 0.0123	0.778 ± 0.0052	0.235 ± 0.015
svmPoly	0.672 ± 0.0085	0.774 ± 0.0057	0.701 ± 0.0081	0.624 ± 0.0085	0.464 ± 0.0132	0.792 ± 0.0064	0.265 ± 0.018
svmRadial	0.679 ± 0.0069	0.794 ± 0.0050	0.697 ± 0.0073	0.617 ± 0.0087	0.452 ± 0.0135	0.790 ± 0.0053	0.249 ± 0.018
xgbLinear	0.718 ± 0.0097	0.833 ± 0.0067	0.714 ± 0.0069	0.631 ± 0.0077	0.470 ± 0.0123	0.804 ± 0.0050	0.285 ± 0.017
xgbTree	0.719 ± 0.0103	0.832 ± 0.0063	0.719 ± 0.0072	0.638 ± 0.0073	0.482 ± 0.0112	0.807 ± 0.0056	0.301 ± 0.016

Rituximab

Model	MAE	RMSE	R^2
gbm	0.290 ± 0.0030	0.360 ± 0.0033	0.0417 ± 0.0051
glmnet	0.333 ± 0.0045	0.417 ± 0.0048	0.0599 ± 0.0065
rf	0.261 ± 0.0013	0.318 ± 0.0014	0.0932 ± 0.0069
SL	0.315 ± 0.0039	0.395 ± 0.0043	0.0597 ± 0.0063
svmPoly	0.310 ± 0.0036	0.391 ± 0.0040	0.0654 ± 0.0067
svmRadial	0.263 ± 0.0020	0.336 ± 0.0018	0.0504 ± 0.0049
xgbLinear	0.255 ± 0.0028	0.336 ± 0.0031	0.1313 ± 0.0087
xgbTree	0.293 ± 0.0036	0.364 ± 0.0043	0.0534 ± 0.0074

Data are presented as mean +/- sem for performance measured using 10x10-fold nested CV with 25 repeats. Etanercept and tocilizumab models were trained with a binary outcome (DAS28-ESR < 3.2) and binary performance metrics are shown. AUC = Area under receiver operating characteristic (ROC) curve, AUC.PR = area under precision-recall curve, F1 = F1 score (positive class is shown), MCC = Matthews correlation coefficient. Rituximab models were trained as an ordinal regression model for DAS28-ESR outcome at 16 weeks. The final selected rituximab model (xgbLinear) was converted to a binary model for comparison with other models in Fig. 5.

Supplementary Table 5. Confusion matrices for prediction models trained in STRAP

**Etanercept**	Reference				
Predicted	Non-responder	Responder	Response rate		
Non-responder	23	9	28%	Accuracy	0.716
Responder	10	25	71%	Balanced accuracy	0.716

**Tocilizumab**	Reference				
Predicted	Non-responder	Responder	Response rate		
Non-responder	12	8	40%	Accuracy	0.754
Responder	9	40	82%	Balanced accuracy	0.702

**Rituximab**	Reference				
Predicted	Non-responder	Responder	Response rate		
Non-responder	39	7	15%	Accuracy	0.736
Responder	12	14	54%	Balanced accuracy	0.716

Supplementary Table 6. Confusion matrices for STRAP prediction models tested in R4RA

**Tocilizumab**	Reference				
Predicted	Non-responder	Responder	Response rate		
Non-responder	33	18	35%	Accuracy	0.692
Responder	2	12	86%	Balanced accuracy	0.752

**Rituximab**	Reference				
Predicted	Non-responder	Responder	Response rate		
Non-responder	43	7	14%	Accuracy	0.765
Responder	9	9	50%	Balanced accuracy	0.680

Supplementary Table 7. Confusion matrices for STRAP & R4RA pooled prediction models

**Tocilizumab**	Reference				
Predicted	Non-responder	Responder	Response rate		
Non-responder	36	17	32%	Accuracy	0.785
Responder	20	61	75%	Balanced accuracy	0.713

**Rituximab**	Reference				
Predicted	Non-responder	Responder	Response rate		
Non-responder	98	21	18%	Accuracy	0.750
Responder	5	16	76%	Balanced accuracy	0.692

Supplementary Table 8. Relationship between anti-CCP and rheumatoid factor seropositivity and clinical response following 16 weeks of biologic therapy in STRAP and R4RA

STRAP

	Etanercept			Tocilizumab			Rituximab		
**ACPA status**	Negative	Positive	P-value	Negative	Positive	P-value	Negative	Positive	P-value
	n=14	n=53		n=15	n=54		n=12	n=60	
**Endpoint**									
ACR20	5 (36%)	33 (62%)	0.14	12 (80%)	39 (72%)	0.78	4 (33%)	40 (67%)	0.066
ACR50	2 (14%)	25 (47%)	0.054	8 (53%)	29 (54%)	1	1 (8%)	18 (30%)	0.23
CDAI < 50%	5 (36%)	39 (74%)	0.019	11 (73%)	37 (69%)	0.97	4 (33%)	34 (57%)	0.25
Target DAS28-ESR < 3.2	4 (29%)	30 (57%)	0.12	12 (80%)	36 (67%)	0.5	2 (17%)	19 (32%)	0.49
Target DAS28-CRP < 3.2	3 (21%)	32 (60%)	0.022	8 (53%)	33 (61%)	0.81	2 (17%)	26 (43%)	0.16
EULAR DAS28-ESR good/mod vs non	4 (29%)	30 (57%)	0.12	12 (80%)	36 (67%)	0.5	2 (17%)	15 (25%)	0.8
EULAR DAS28-CRP good/mod vs non	3 (21%)	34 (64%)	0.011	9 (60%)	32 (59%)	1	2 (17%)	24 (40%)	0.23

STRAP & R4RA

	Tocilizumab			Rituximab		
**ACPA status**	Negative	Positive	P-value	Negative	Positive	P-value
	n=30	n=104		n=26	n=114	
**Endpoint**						
CDAI < 50%	17 (57%)	68 (65%)	0.51	10 (38%)	57 (50%)	0.4
Target DAS28-ESR < 3.2	19 (63%)	59 (57%)	0.66	5 (19%)	32 (28%)	0.5
Target DAS28-CRP < 3.2	15 (50%)	56 (54%)	0.87	8 (31%)	42 (37%)	0.72
EULAR DAS28-ESR good/mod vs non	19 (63%)	55 (53%)	0.42	18 (69%)	74 (65%)	0.85
EULAR DAS28-CRP good/mod vs non	20 (67%)	57 (55%)	0.34	15 (58%)	66 (58%)	1

STRAP

	Etanercept			Tocilizumab			Rituximab		
**RF status**	Negative	Positive	P-value	Negative	Positive	P-value	Negative	Positive	P-value
	n=19	n=48		n=23	n=46		n=20	n=52	
**Endpoint**									
ACR20	9 (47%)	29 (60%)	0.49	17 (74%)	34 (74%)	1	10 (50%)	34 (65%)	0.35
ACR50	7 (37%)	20 (42%)	0.93	13 (57%)	24 (52%)	0.93	2 (10%)	17 (33%)	0.097
CDAI < 50%	10 (53%)	34 (71%)	0.26	18 (78%)	30 (65%)	0.41	6 (30%)	32 (62%)	0.033
Target DAS28-ESR < 3.2	8 (42%)	26 (54%)	0.54	17 (74%)	31 (67%)	0.78	2 (10%)	19 (37%)	0.054
Target DAS28-CRP < 3.2	9 (47%)	26 (54%)	0.82	15 (65%)	26 (57%)	0.66	3 (15%)	25 (48%)	0.021
EULAR DAS28-ESR good/mod vs non	8 (42%)	26 (54%)	0.54	17 (74%)	31 (67%)	0.78	1 (5%)	16 (31%)	0.046
EULAR DAS28-CRP good/mod vs non	10 (53%)	27 (56%)	1	16 (70%)	25 (54%)	0.34	1 (5%)	25 (48%)	0.0017

STRAP & R4RA

	Tocilizumab			Rituximab		
**RF status**	Negative	Positive	P-value	Negative	Positive	P-value
	n=44	n=90		n=36	n=104	
**Endpoint**						
CDAI < 50%	31 (70%)	54 (60%)	0.32	13 (36%)	54 (52%)	0.15
Target DAS28-ESR < 3.2	27 (61%)	51 (57%)	0.74	6 (17%)	31 (30%)	0.19
Target DAS28-CRP < 3.2	25 (57%)	46 (51%)	0.66	9 (25%)	41 (39%)	0.18
EULAR DAS28-ESR good/mod vs non	26 (59%)	48 (53%)	0.66	20 (56%)	72 (69%)	0.2
EULAR DAS28-CRP good/mod vs non	27 (61%)	50 (56%)	0.65	18 (50%)	63 (61%)	0.36

P-values were obtained using a two-sided Chi-Square test.

STRAP Collaborative Group

Ahmed Zayat
Alberto Cauli
Ana Rita Machado
Andrea Cuervo
Andrew Filer
Anne Barton
Arthur G. Pratt
Arti Mahto
Bhaskar Dasgupta
Charlotte Rawlings
Chijioke Mosanya
Christopher D. Buckley
Christopher J. Edwards
Chris Holroyd
Claire Gorman
Deborah Maskall
Désirée van der Heijde
Deepak Jaydon
Edyta Jaworska
Elisa Gremese
Ernest Choy
Frances Humby
Francesco Carlucci
Georgina Thorburn
Gina Tan
Gloria Lliso-Ribera
Hasan Rizvi
Hector Chinoy
Iain McInnes
James Galloway
Joanna Peel
João Eurico Fonseca
Joaquim P. Pereira
John D. Isaacs
Juan D. Cañete
Julio Ramírez
Laurent Meric de Bellefon
Louise Warren
Mary Githinji
Maya H. Buch
Mattia Congia
Michael R. Ehrenstein
Michele Bombardieri
Nagui Gendi
Neal Millar
Neil D. McKay
Nora Ng
Nirupam Purkayastha
Patrick Durez
Pauline Ho
Peter C. Taylor
Peter Sasieni
Pier Paolo Sainaghi
Rakhi Seth
Raquel Celis
Rebecca Hands-Greenwood
Richard Stratton
Robert Landewé
Sabrina Raizada
Simone Perniola
Stefano Alivernini
Stefano Marcia
Stefano Marini
Stephen Kelly
Vasco Romão
