# Supplementary material for: Deep molecular profiling of synovial biopsies in the STRAP trial identifies signatures predictive of treatment response to biologic therapies in rheumatoid arthritis
Source: Nat Commun. 2025 Jul 2;16:5374. doi: 10.1038/s41467-025-60987-9 (PMC12223067; doi:10.1038/s41467-025-60987-9)
Supplement: Supplementary file 2 — Description of Additional Supplementary Files [file 41467_2025_60987_MOESM2_ESM.pdf]

Description of Additional Supplementary Files

Supplementary Data 1: Synovial DEG signatures of response to biologics at baseline in RA

Table of differential gene expression analysis results using DESeq2 with PC1 as covariate between all ACR20 response vs non-response patients treated with etanercept (Fig. 1a, n=38 responders, n=29 non-responders), tocilizumab (Fig. 1c, n=51 responders, n=18 non-responders) and rituximab (Fig. 1e, n=44 responders, n=28 non-responders). Table contains average normalised count values (baseMean), log_2-transformed fold changes between the two groups (log2FoldChange), Wald test associated p value (pvalue), and FDR corrected p values with Storey's q value adjustment (qvalue). Results are shown in Fig. 1a, c, e.

Supplementary Data 2: Synovial modular signatures of response to biologics at baseline in RA

Modular analysis applying QuSAGE to responders versus non-responders to etanercept, tocilizumab and rituximab. Log_2 fold changes of responders (positive values) and nonresponders (negative values) for blood microarray-based modules and WGCNA modules with p values with adjustment for multiple testing using Storey's q-value adjustment (qvalue). Results are shown in Fig. 1b, d, f.

Supplementary Data 3: Analysis of common molecular signatures of responsiveness/resistance to biologics

Supplementary data for Fig. 2a & b containing a table of differential gene expression analysis results between all ACR20 responder (n=133) and non-responder (n=75) patients, and lists of pathways ($p < 0.05$) associated with up-regulated genes in responders and non-responders.

Supplementary Data 4: Gene clusters identified in the STRAP and R4RA cohorts, with pathway enrichment analysis applied to clusters from STRAP

Supplementary data for Fig. 4c & d containing the genes associated with each cluster identified in the STRAP cohort (cluster 1=983 genes, cluster 2=1420 genes, cluster 3=1008 genes) and in the R4RA cohort (cluster 1=713 genes, cluster 2=968 genes, cluster 3=581 genes). Complete lists of pathways associated with each STRAP cluster (adjusted $p < 0.05$) are also reported.

Supplementary Data 5: Gene list of custom nCounter panel manufactured for this study
