# Supplementary material for: Deep molecular profiling of synovial biopsies in the STRAP trial identifies signatures predictive of treatment response to biologic therapies in rheumatoid arthritis
Source: Nat Commun. 2025 Jul 2;16:5374. doi: 10.1038/s41467-025-60987-9 (PMC12223067; doi:10.1038/s41467-025-60987-9)
Supplement: Supplementary file 8 — Reporting Summary [file 41467_2025_60987_MOESM8_ESM.pdf]

nature portfolio

Corresponding author(s): Myles Lewis
Last updated by author(s): Mar 27, 2025

Reporting Summary

Nature Portfolio wishes to improve the reproducibility of the work that we publish. This form provides structure for consistency and transparency in reporting. For further information on Nature Portfolio policies, see our Editorial Policies and the Editorial Policy Checklist.

Statistics

For all statistical analyses, confirm that the following items are present in the figure legend, table legend, main text, or Methods section.

n/a	Confirmed	
☐	☒	The exact sample size (*n*) for each experimental group/condition, given as a discrete number and unit of measurement
☐	☒	A statement on whether measurements were taken from distinct samples or whether the same sample was measured repeatedly
☐	☒	The statistical test(s) used AND whether they are one- or two-sided<br>*Only common tests should be described solely by name; describe more complex techniques in the Methods section.*
☐	☒	A description of all covariates tested
☐	☒	A description of any assumptions or corrections, such as tests of normality and adjustment for multiple comparisons
☐	☒	A full description of the statistical parameters including central tendency (e.g. means) or other basic estimates (e.g. regression coefficient) AND variation (e.g. standard deviation) or associated estimates of uncertainty (e.g. confidence intervals)
☐	☒	For null hypothesis testing, the test statistic (e.g. *F*, *t*, *r*) with confidence intervals, effect sizes, degrees of freedom and *P* value noted<br>*Give P values as exact values whenever suitable.*
☒	☐	For Bayesian analysis, information on the choice of priors and Markov chain Monte Carlo settings
☒	☐	For hierarchical and complex designs, identification of the appropriate level for tests and full reporting of outcomes
☐	☒	Estimates of effect sizes (e.g. Cohen's *d*, Pearson's *r*), indicating how they were calculated

Our web collection on statistics for biologists contains articles on many of the points above.

Software and code

Policy information about availability of computer code

Data collection	RNA-Sequencing FastQC files were generated on an Illumina HiSeq 4000 instrument (performed by Genewiz, South Plainfield, NJ, USA). RNA-Seq data was processed using Salmon version 0.13.1, Tximport version 1.13.10 and aligned against human transcriptome reference Gencode v29, GRCh38.p12. Nanostring nCounter data was processed using nanoStringNCTools version 1.6.0.
Data analysis	R version 4.3.0<br>R packages: DESeq2 version 1.34.0, nestedcv version 0.7.9, QuSAGE version 2.30.0, enrichR version 3.2, edgeR version 4.2.0, ComplexHeatmap version 2.14.0, SVA version 3.52.0, shiny server version 1.5.16, plotly version 4.9.3.<br>The nestedcv R package used to build and test the machine learning models is publicly available for installation from the CRAN R repository (DOI: 10.32614/CRAN.package.nestedcv). The source code is also available on GitHub at https://github.com/myles-lewis/nestedcv. Scripts used for figure generation, model building and performance testing are available from https://github.com/EMR-bioinformatics/STRAP.

For manuscripts utilizing custom algorithms or software that are central to the research but not yet described in published literature, software must be made available to editors and reviewers. We strongly encourage code deposition in a community repository (e.g. GitHub). See the Nature Portfolio guidelines for submitting code & software for further information.

Data

Policy information about availability of data

All manuscripts must include a data availability statement. This statement should provide the following information, where applicable:

- Accession codes, unique identifiers, or web links for publicly available datasets
- A description of any restrictions on data availability
- For clinical datasets or third party data, please ensure that the statement adheres to our policy

The datasets generated during and analysed during the current study are available on an interactive web interface that allows direct data exploration (https://strap.hpc.qmul.ac.uk/). A searchable interface allows users to examine relationships between individual synovial gene transcript levels and histological and clinical parameters, and clinical response at 16 weeks. The website was constructed using R shiny server 1.5.16, with interactive plots generated with R plotly 4.9.3. RNA-Seq data is available at ArrayExpress accession ID E-MTAB-13733.

Research involving human participants, their data, or biological material

Policy information about studies with human participants or human data. See also policy information about sex, gender (identity/presentation), and sexual orientation and race, ethnicity and racism.

Reporting on sex and gender	Participants' sex was collected from hospital records.
Reporting on race, ethnicity, or other socially relevant groupings	Self reported ethnicity was reported with the baseline characteristics of the main clinical trials STRAP and STRAP EU as published in Rivellese F et al, Lancet Rheumatology 2023, 5: e648–59.
Population characteristics	Patients will be recruited with active RA:<br>1. 2010 ACR / EULAR Rheumatoid Arthritis classification criteria for a diagnosis of RA<br>2. Patient with DMARD failure eligible for anti-TNF-α therapy as per UK NICE guidelines<br>3. Patients must have a minimum of 3 swollen joints – the joint selected for biopsy and a minimum of 2 from 28 joint count set, as assessed at biopsy visit<br>4. Selected joint for biopsy must be minimum grade 2 synovial thickening, as assessed at the biopsy visit<br>5. 18 years of age and over<br>6. Patients must be capable of giving informed consent and the consent must be obtained prior to any screening procedures<br>* The ACR/EULAR classification for a diagnosis of RA could have been at any time in the patient's disease history; the score does not need to be 6 or more at screening.
Recruitment	Patients were identified through rheumatology outpatient clinics at participating sites at 26 centres across the UK and EU.
Ethics oversight	UK ethics committee approval MREC 14/WA/1209.<br>Comité d'Ethique Hospitalo-Facultaire Saint-Luc, Bruxelles, Belgium (18/3/2019).<br>Comitato Etico Interaziendale, AOU Maggiore della Crita, Novara, Italy (6/9/2019).<br>Comissão de Ética para a Investigação clinica, Lisbon, Portugal (25/9/2019).<br>Comité de ética de la investigacion con medicamentos, Barcelona, Spain (28/9/2018).

Note that full information on the approval of the study protocol must also be provided in the manuscript.

Field-specific reporting

Please select the one below that is the best fit for your research. If you are not sure, read the appropriate sections before making your selection.

[x] Life sciences [ ] Behavioural & social sciences [ ] Ecological, evolutionary & environmental sciences

For a reference copy of the document with all sections, see nature.com/documents/nr-reporting-summary-flat.pdf

Life sciences study design

All studies must disclose on these points even when the disclosure is negative.

Sample size	Sample size was calculated for the original clinical trial as follows, as reported in the Lancet Rheumatology paper for the main study. A sample size of 96 B cell-poor patients was planned to achieve 80% power and 126 B cell-poor patients to achieve 90% power to test the difference in response rates between treatment groups for the primary endpoint. This was based on assuming a response rate of 30% for the rituximab group and 60% for etanercept and tocilizumab groups, with a two-sided 5% type 1 error rate and a dropout rate of 10%.
Data exclusions	For the main clinical trial, the primary and secondary analyses were based on the intention-to-treat population, defined as all randomly assigned patients. Following RNA-Seq quality control, with Principal Component Analysis (PCA), 5 baseline and 3 follow-up, in total 8 samples were excluded due to poor mapping rate that was originated from low RNA quality (Supplementary Fig. 1a). Thus RNA-Seq data from 208 patients were available for subsequent analysis at baseline (65 samples at later time points are not analysed here).
Replication	Two of the machine learning models built on STRAP data were validated in the independent R4RA cohort. In the R4RA trial, patients (n=164)

	were randomised to tocilizumab or rituximab (not etanercept or anti-TNF) [Rivellese et al 2022, Nature medicine]. Therefore only these two models were tested in R4RA. Of the 164 patients randomised on entry to the R4RA trial, good quality RNA-Seq was available on n=133 post-QC.
Randomization	Before randomisation, participants were stratified according to synovial histopathology (B cell rich, B cell poor, or unknown B cell status) and methotrexate use, and subsequently randomly assigned (1:1:1) to rituximab, etanercept, or tocilizumab using hierarchical dynamic randomisation. Randomisation was performed by the STRAP Trial Office within the Barts Clinical Trials Unit (London, UK). The randomisation list was prepared by the trial statistician, and the application codes of hierarchical dynamic randomisation were securely embedded with the application code so that it was not accessible to end users in order to ensure concealment.
Blinding	The named joint assessor was masked to study drug allocation, and all staff at the recruiting sites were masked to the B cell classification throughout the study.

Reporting for specific materials, systems and methods

We require information from authors about some types of materials, experimental systems and methods used in many studies. Here, indicate whether each material, system or method listed is relevant to your study. If you are not sure if a list item applies to your research, read the appropriate section before selecting a response.

Materials & experimental systems

n/a	Involved in the study
☐	☒ Antibodies
☒	☐ Eukaryotic cell lines
☒	☐ Palaeontology and archaeology
☒	☐ Animals and other organisms
☒	☐ Clinical data
☒	☐ Dual use research of concern
☒	☐ Plants

Methods

n/a	Involved in the study
☒	☐ ChIP-seq
☒	☐ Flow cytometry
☒	☐ MRI-based neuroimaging

Antibodies

Antibodies used	The following primary antibodies were used: CD79A (clone JCB117, Dako), CD3 (clone F7.238, Dako), CD20 (clone L26, Dako), CD68 (clone KP1, Dako) and CD138 (clone MI15, Dako).
Validation	All antibodies have been validated in several previous papers (Lewis M et al 2019, Cell Reports 28, 2455–2470; Rivellese F et al 2022, Nature medicine, doi.org/10.1038/s41591-022-01789-0).

Plants

Seed stocks	*Report on the source of all seed stocks or other plant material used. If applicable, state the seed stock centre and catalogue number. If plant specimens were collected from the field, describe the collection location, date and sampling procedures.*
Novel plant genotypes	*Describe the methods by which all novel plant genotypes were produced. This includes those generated by transgenic approaches, gene editing, chemical/radiation-based mutagenesis and hybridization. For transgenic lines, describe the transformation method, the number of independent lines analyzed and the generation upon which experiments were performed. For gene-edited lines, describe the editor used, the endogenous sequence targeted for editing, the targeting guide RNA sequence (if applicable) and how the editor was applied.*
Authentication	*Describe any authentication procedures for each seed stock used or novel genotype generated. Describe any experiments used to assess the effect of a mutation and, where applicable, how potential secondary effects (e.g. second site T-DNA insertions, mosiacism, off-target gene editing) were examined.*
